# Supplementary material for: The E2F1-HMGCR axis promotes ferroptosis resistance in immune refractory tumor cells
Source: Nat Commun. 2025 Dec 3;16:10849. doi: 10.1038/s41467-025-66282-x (PMC12675509; doi:10.1038/s41467-025-66282-x)
Supplement: Supplementary file 1 — Supplementary Information [file 41467_2025_66282_MOESM1_ESM.pdf]

Supplemental Figures, table and Figure legends

The E2F1-HMGCR axis promotes ferroptosis resistance in immune refractory tumor cells

Sung Wook Son, et al

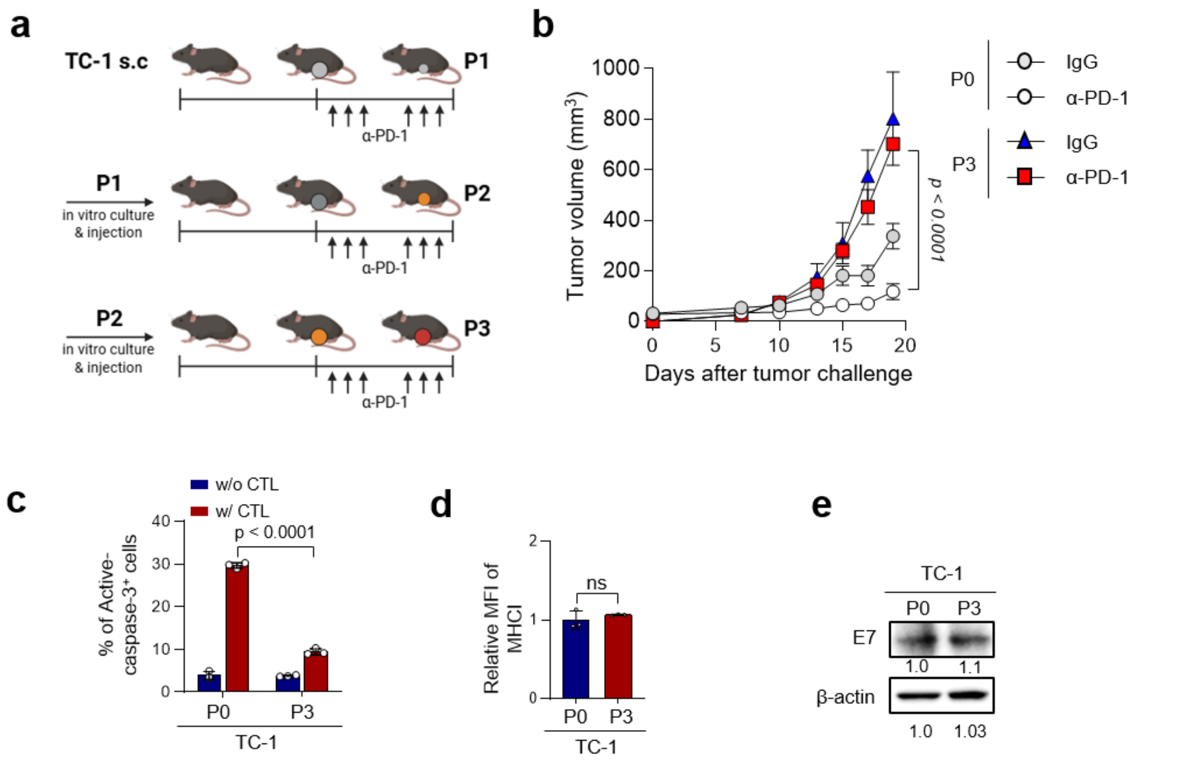

Supplementary Fig. 1 Establishment and characterization of ICB-refractory TC-1 tumor model. a Schematic illustration of the establishment of mouse anti-PD-1 refractory TC-1 tumor model. This image was created in BioRender. Lee, H. (http://biorender.com/x5el3li). **b** Tumor volume of mice inoculated with TC-1 P0 or P3 cells treated with IgG or α-PD-1. **c** Apoptotic CFSE-labeled tumor cells exposed to tumor-specific CTLs were determined by flow cytometric analysis of active-caspase-3. **d** MHC class I expression on TC-1 P0 and P3 cells measured by flow cytometry. **e** Levels of E7 protein, a tumor antigen of TC-1 cells, determined by Western blot. β-actin was included as an internal loading control. For the *in vivo* experiments, 10 mice from each group were used, and randomly selected 7 samples were analyzed. All *in vitro* experiments were performed in triplicate. The data represent the mean ± SD. The *p*-values by two-way ANOVA **b, c** and unpaired, two-tailed Student's t test **d** are indicated. NS, not significant. Source data are provided as a Source data file.

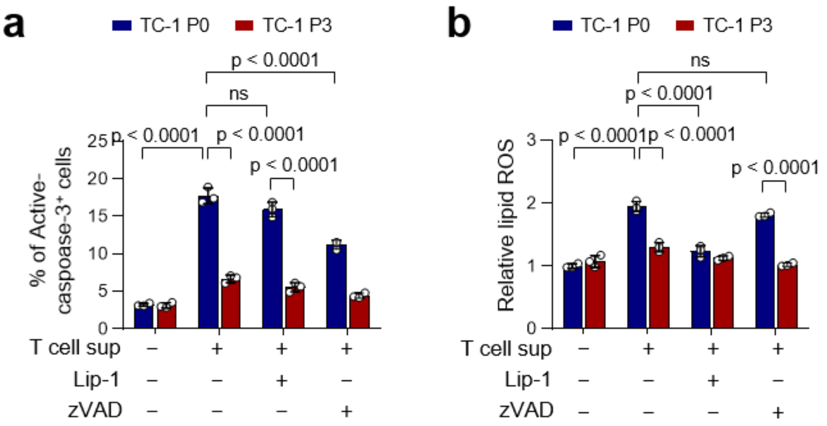

Supplementary Fig. 2 TC-1 P3 cells develop resistance to CTL-induced apoptotic and ferroptotic cell death. a and b TC-1 P0 and P3 cells were incubated with CTL- derived supernatant with or without zVAD or Lip-1. The active-caspase-3 **a** and relative lipid ROS **b** were measured by flow cytometry. Cells were then co-treated with the ferroptosis inhibitor Lip-1 or the apoptosis inhibitor zVAD, and ferroptosis (characterized by lipid peroxidation)[1] and apoptosis (marked by active caspase-3) were evaluated. All *in vitro* experiments were performed in triplicate. The data represent the mean ± SD. The p values by one-way ANOVA. Source data are provided as a Source data file.

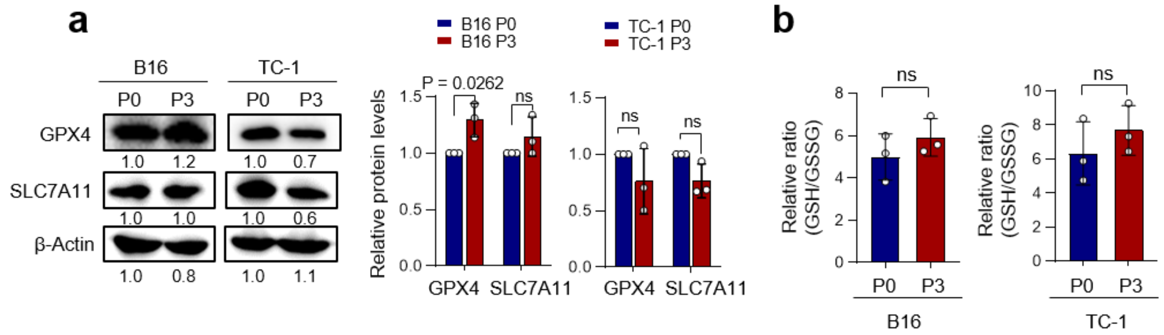

Supplementary Fig. 3 The protein levels of GPX4 and SLC7A11, as well as GPX4 activity, remain unchanged in P3 compared to P0. a Levels of GPX4, SLC7A11 and β-Actin were proved by Western blot. β-Actin was included as an internal loading control. Numbers below blot images indicate the expression as measured by fold change. Graph depicts the experimental quantitation based on at least three independent experiments. **b** Relative GSH/GSSG ratio in B16 or TC-1 P0 and P3 cells. The glutathione (GSH) to glutathione disulfide (GSSG) ratio is a well-established and commonly employed indicator of GPX4 activity, as recommended in ferroptosis evaluation guidelines[2,3]. All *in vitro* experiments were performed in triplicate. The data represent the mean ± SD. The *p*-values by unpaired, two-tailed Student's t test **a and b** are indicated. NS, not significant. Source data are provided as a Source data file.

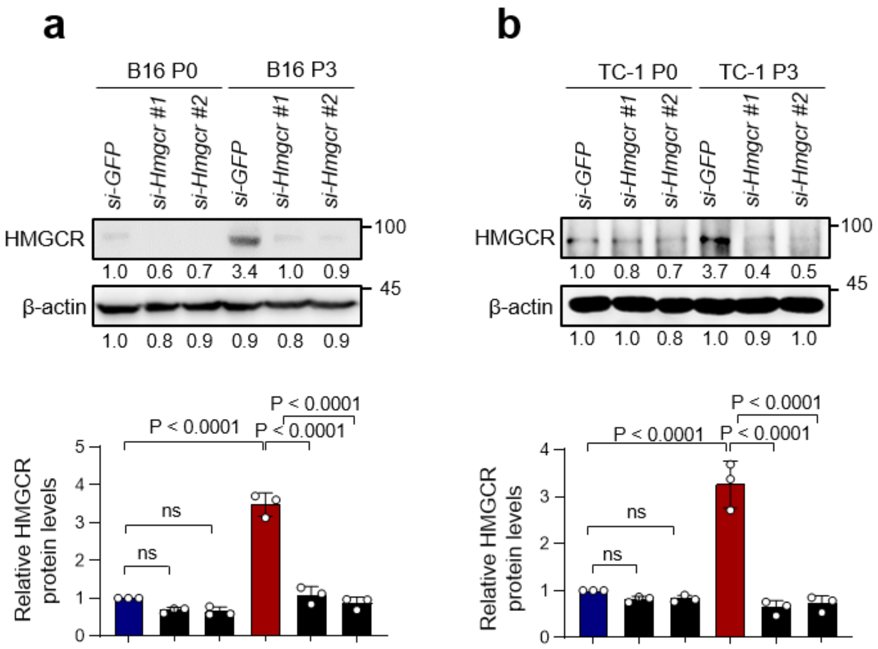

Supplementary Fig. 4 The protein levels of HMGCR in immune refractory tumor cells following treatment with *siGFP*, *siHMGCR #1 or siHMGCR #2*. a and b Levels of HMGCR protein in B16 P0 and P3 cells **a** and in TC-1 P0 and P3 cells **b** were measured by Western blot. Numbers below blot images indicate the expression as measured by fold change. Graph depicts the experimental quantitation based on at least three independent experiments. β-actin was included as an internal loading control. The data represent the mean ± SD. The *p*-values by unpaired, two-tailed Student's t test **a, b** are indicated. NS, not significant. Source data are provided as a Source data file.

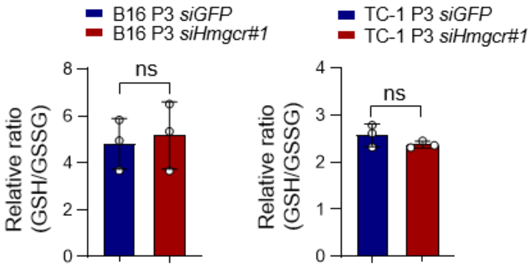

Supplementary Fig. 5 Depletion of HMGCR in P3 cells does not affect GPX4 activity. Relative GSH/GSSG ratio in B16 or TC-1 P0 and P3 cells following treatment with siGFP, siHMGCR #1. All *in vitro* experiments were performed in triplicate. The *p*-values by unpaired, two-tailed Student's t test are indicated. ns, not significant. Source data are provided as a Source data file.

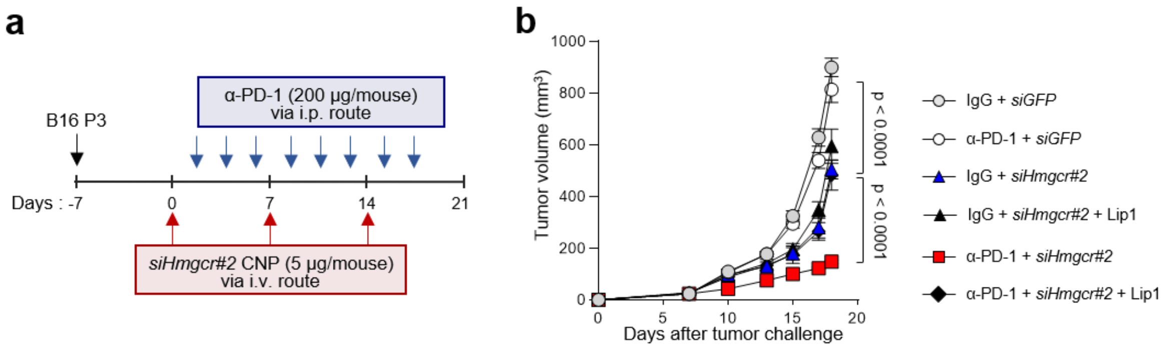

Supplementary Fig. 6 Silencing of HMGCR reverses immune-refractory features by inducing the anti-PD-1-mediated ferroptotic cell death. a Schematic of the therapy regimen in mice implanted with B16 P3 cells. **b** Tumor growth during 18 days after mice inoculated with B16 P3 then treated with the indicated reagents. B16 P3 tumor–bearing mice were administered siGFP or siHmgcr#2 with Lip-1 or anti–PD-1 or anti-PD-1 plus Lip-1 as indicated. For the in vivo experiments, 6 mice from each group were used. The error bars represent mean ± SD. The p values by two-way ANOVA are indicated. Source data are provided as a Source data file.

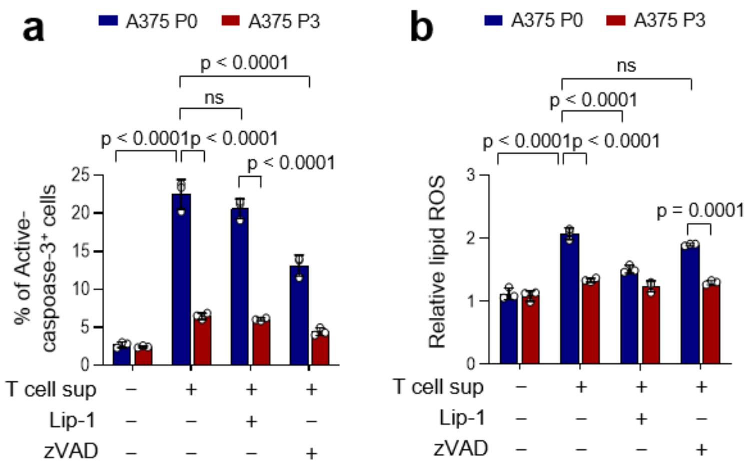

Supplementary Fig. 7 A375 P3 cells exhibit resistance to both apoptosis and ferroptosis under CTL-mediated cytotoxic conditions. a and b A375 P0 and P3 cells were incubated with CTL-derived supernatant with or without zVAD or Lip-1. The active-caspase-3 **a** and relative lipid ROS **b** was measured by flow cytometry. All in vitro experiments were performed in triplicate. The data represents the mean ± SD. The p values by one-way ANOVA. Source data are provided as a Source data file.

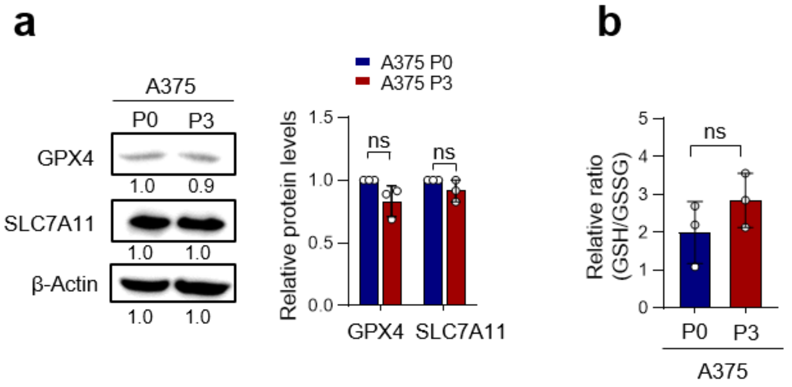

Supplementary Fig. 8 The protein levels of GPX4 and SLC7A11, as well as GPX4 activity, remain unchanged in A375 P3 compared to P0. a Levels of GPX4, SLC7A11 and β-Actin were proved by Western blot. β-Actin was included as an internal loading control. Numbers below blot images indicate the expression as measured by fold change. Graph depicts the experimental quantitation based on at least three independent experiments. **b** Relative GSH/GSSG ratio in A375 P0 and P3 cells. The *p*-values by unpaired, two-tailed Student's t test **a and b** are indicated. ns, not significant. Source data are provided as a Source data file.

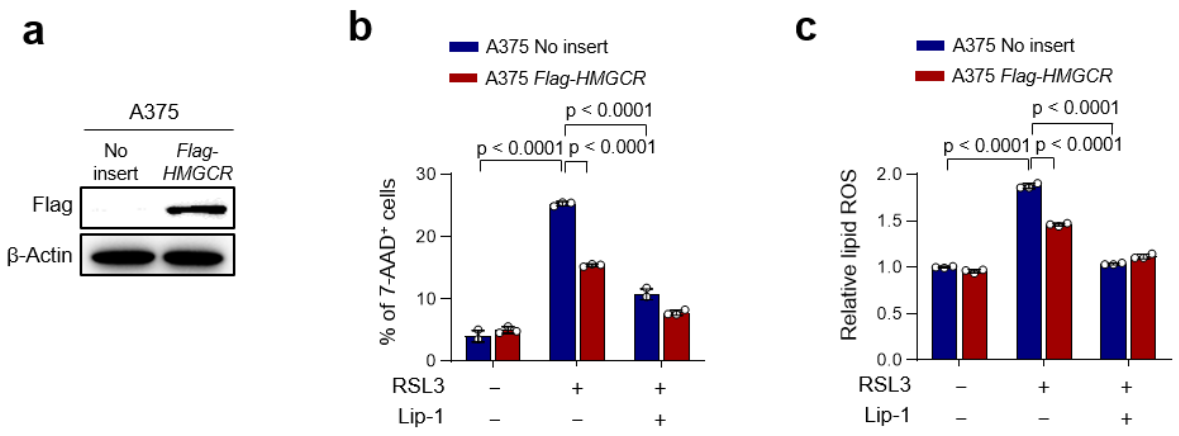

Supplementary Fig. 9 Overexpression of HMGCR induces ferroptosis resistance in A375 P0 cells. **a** A375 cells were transfected with empty vector (no insert), FLAG-HMGCR wild type (HMGCR WT). β-actin was included as an internal loading control. **b and c** The 7AAD$^+$ cells **b** and relative lipid ROS **c** were measured by flow cytometry. All in vitro experiments were performed in triplicate. The data represent the mean ± SD. The p values by one-way ANOVA. Source data are provided as a Source data file.

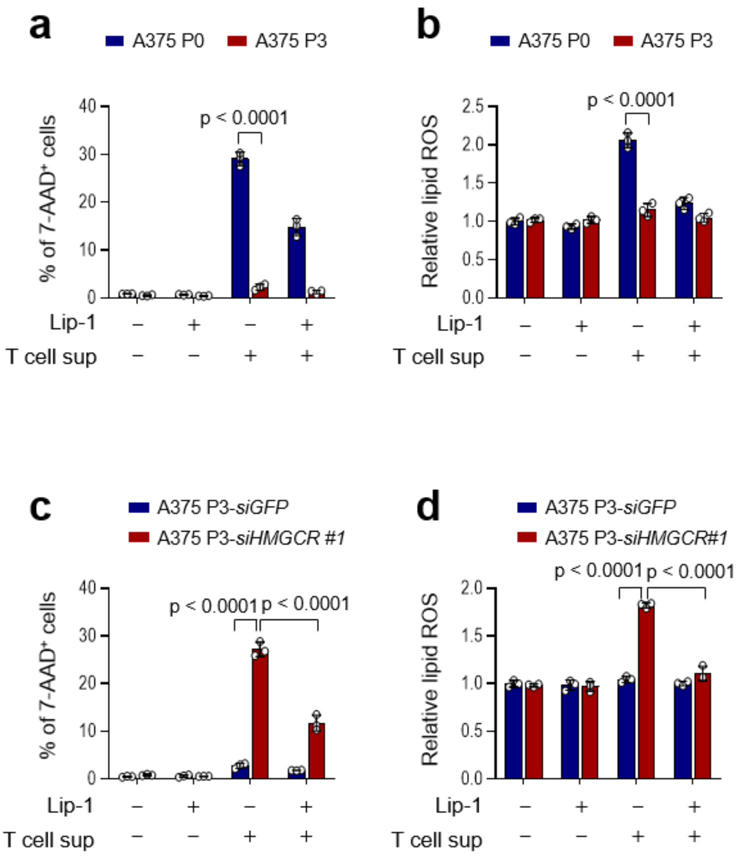

Supplementary Fig. 10 HMGCR silencing sensitizes A375 P3 cells to CTL supernatant-induced ferroptosis. a and b Tumor cells were incubated with the supernatant from tumor-specific CTLs with or without Lip-1. **c and d** A375 P3 cells were transfected with *siGFP* targeting GFP or *siHMGCR#1* targeting HMGCR. The 7AAD+ cells **a, c** and relative lipid ROS **b, d** were measured by flow cytometry. All *in vitro* experiments were performed in triplicate. The data represent the mean ± SD. The p values by one-way ANOVA. Source data are provided as a Source data file.

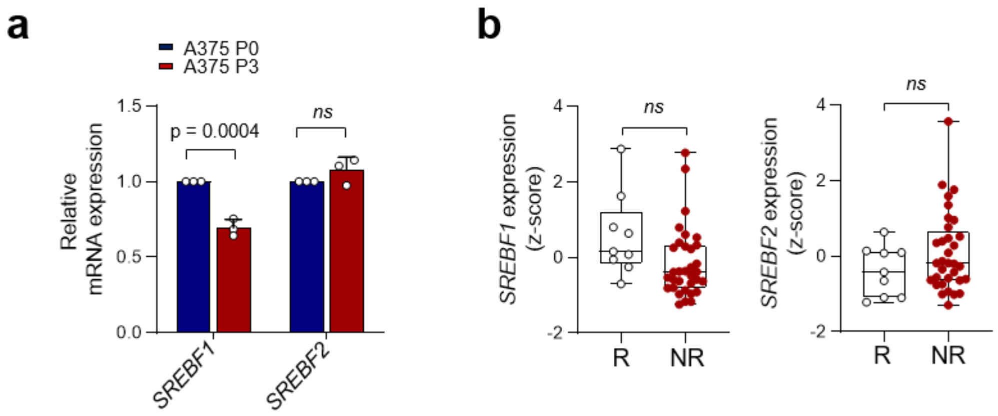

Supplementary Fig. 11 SREBF expression shows no correlation with A375 P3 phenotype or ICB response a The qPCR analysis of levels of SREBP1 and SREBP2 mRNAs in indicated A375 P0 and P3 cells. **b** Comparisons of expressions levels of SREBP1 and SREBP2 in responder (R, n = 9) and non-responder (NR, n = 33). Differences in cytotoxicity or expression level were statistically tested using the unpaired, two-tailed Student's t test **a and b**, ns, not significant. All *in vitro* experiments were performed in triplicate. The error bars represent mean ± SD. Source data are provided as a Source data file.

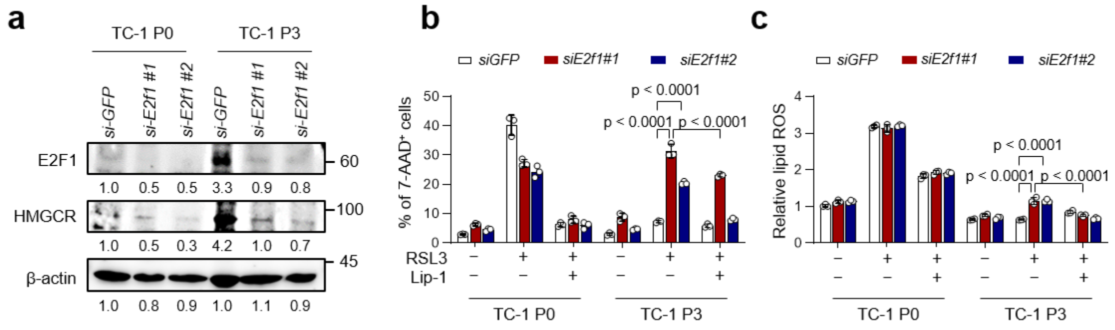

Supplementary Fig. 12 Loss of E2F1 sensitizes TC-1 P3 cells to RSL3-induced ferroptosis a Levels of E2F1 and HMGCR proteins in TC-1 P0 and P3 cells transfected with *siGFP, siE2F1#1 or siE2F1#2* detected by Western blot. β-actin was included as an internal loading control. **b and c** The percentage of 7-AAD$^+$ cells **b** and the level of lipid ROS **c** in TC-1 P0 and P3 cells treated with the indicated agents measured by flow cytometry. The data are representative of those from 3 independent experiments with triplicate. The error bars represent mean ± SD. The p values by one-way ANOVA. Source data are provided as a Source data file.

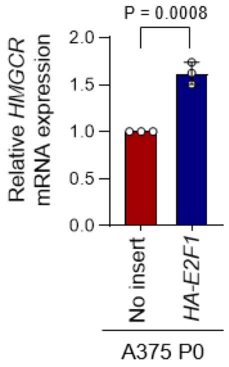

Supplementary Fig. 13 E2F1 induces HMGCR expression in A375 P0 cells. Levels of the HMGCR mRNA in A375 P0 cells transfected with an empty plasmid or a HA-E2F1-expressing plasmid were determined by qRT-PCR. The data are representative of those from 3 independent experiments with triplicate. The error bars represent mean ± SD. The p values by unpaired, two-tailed Student's t test. Source data are provided as a Source data file.

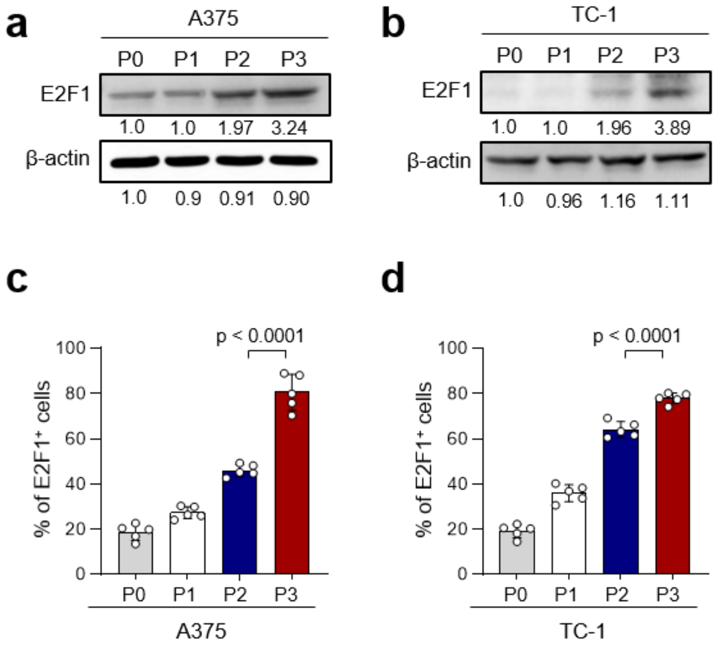

Supplementary Fig. 14 Quantification of E2F1 expression in tumor cells at different stages of selection with CTL or ICB. a and b E2F1 protein levels in A375 cells **a** or TC-1 cells **b** at various stages of immune-resistance were determined by Western blot. **c and d** The percentage of E2F1$^+$ cells were analyzed by flow cytometry. All *in vitro* experiments were performed in triplicate. The error bars represent mean ± SD. The p values by one-way ANOVA **c, d** are indicated. Source data are provided as a Source data file

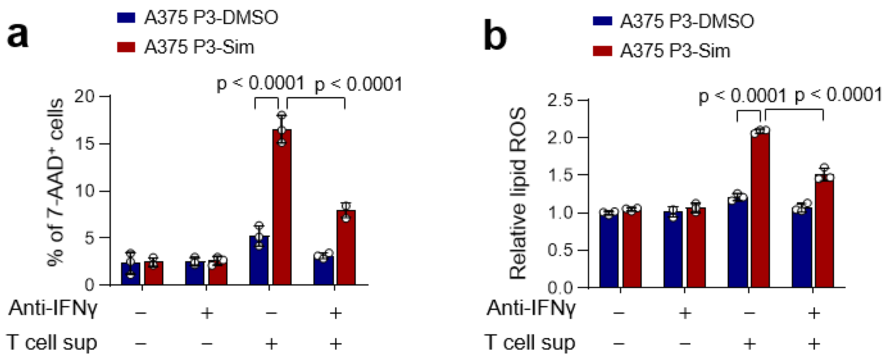

Supplementary Fig. 15 Simvastatin sensitizes A375 P3 cells to CTL supernatant-induced ferroptosis via IFNγ signaling. a and b A375 cells were incubated with the CTLs with or without Anti-IFNγ and treated with DMSO or Sim targeting HMGCR. The 7AAD$^+$ cells **a** and relative lipid ROS **b** were measured by flow cytometry. All *in vitro* experiments were performed in triplicate. The data represent the mean ± SD. The p values by one-way ANOVA. Source data are provided as a Source data file.

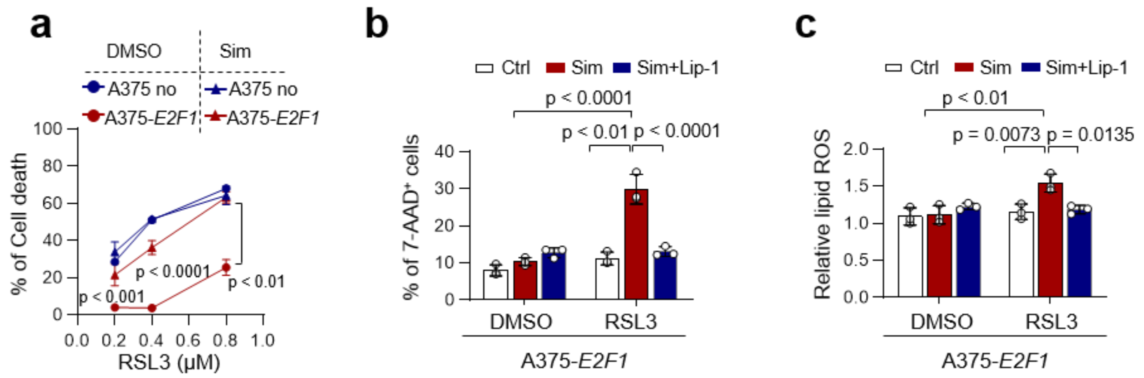

Supplementary Fig. 16 RSL3 combined with simvastatin impairs ferroptosis resistance of A375-E2F1 cells. a The percentages of cell death in A375 no insert and A375 HA-E2F1 cells treated with indicated concentrations of RSL3 with or without Sim (1μM) for 24h. The percentage of cell death was determined by trypan blue exclusion assay. **b and c** The percentage of 7AAD$^+$ tumor cells **b** or relative lipid ROS **c** in A375 E2F1 cells measured by flow cytometry. The data are representative of those from 3 independent experiments with triplicate. The error bars represent mean ± SD. The p values by unpaired, two-tailed Student's t test **a** and one-way ANOVA **b, c** are indicated. Source data are provided as a Source data file.

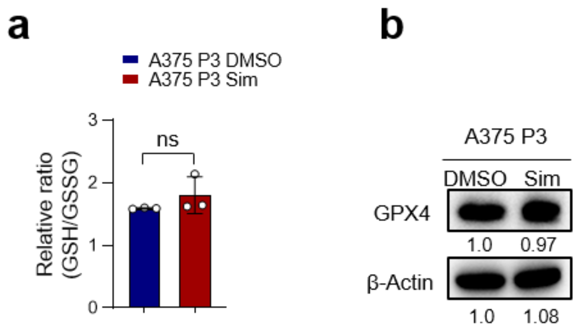

Supplementary Fig. 17 Simvastatin treatment in A375 P3 does not affect the activity and protein levels of GPX4. a Relative GSH/GSSG ratio in A375 P3 cells following treatment with simvastatin. **b** Levels of GPX4 and β-Actin were proved by Western blot. β-Actin was included as an internal loading control. All *in vitro* experiments were performed in triplicate. The error bars represent mean ± SD. The *p*-values by unpaired t-test **a** are indicated. NS, not significant. Source data are provided as a Source data file.

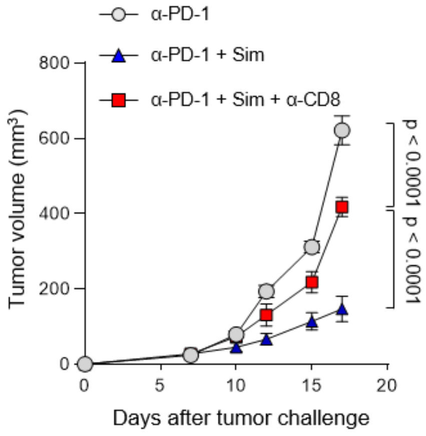

Supplementary Fig. 18 Simvastatin enhances the anti-tumor efficacy of anti–PD-1 therapy in a CD8$^+$ T cell–dependent manner. Tumor growth in mice inoculated with B16 P3 cells and treated with the indicated reagents. For the in vivo experiments, 10 mice from each group were used, and 6 samples were analyzed randomly selected. The p values by two-way ANOVA are indicated. The error bars represent mean ± SD. Source data are provided as a Source data file.

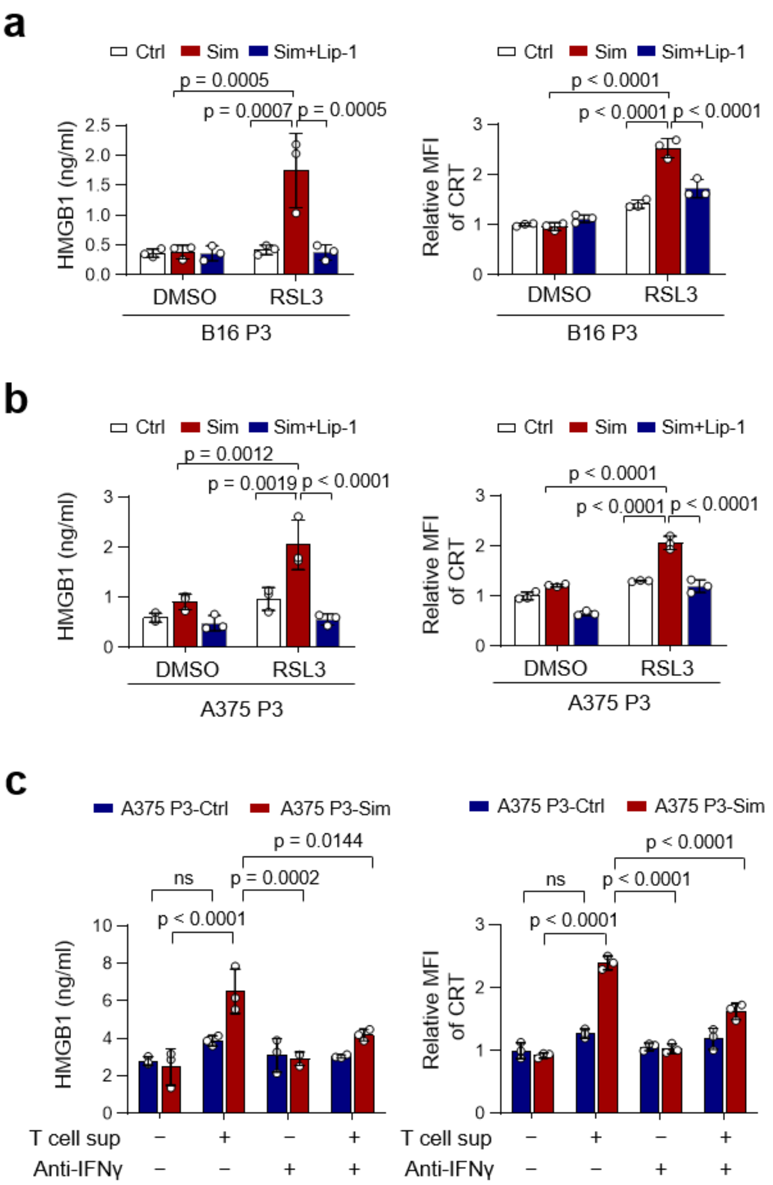

Supplementary Fig. 19 Combined with simvastatin and RSL3 induce DAMP release in immune-refractory tumor cells. a and b the level of HMGB1 and calreticulin (CRT) in B16 P3 cells and in A375 P3 cells treated for 48h with RSL3 and Sim (1 μM) with or without Lip-1 (1 μM) for an additional 20h. The level of HMGB1 measured using HMGB1 enzyme-linked immunosorbent assay (ELISA) kit. Relative MFI of CRT in B16 P3 cells and in A375 P3 cells as assessed using flow cytometry. **c** Tumor cells were incubated with the supernatant from tumor-specific CTLs with or without Anti-IFNγ. All *in vitro* experiments were performed in triplicate. The error bars represent mean ± SD. The p values by one-way ANOVA **a-c** are indicated. Source data are provided as a Source data file.

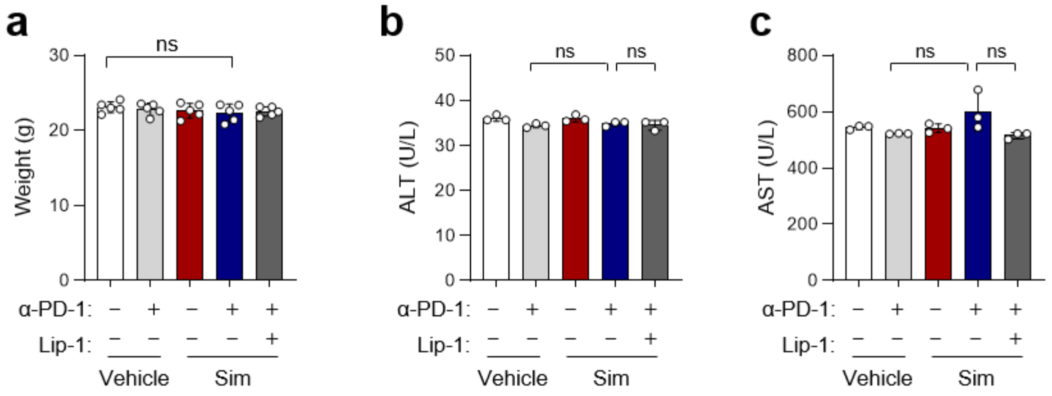

Supplementary Fig. 20 Simvastatin does not affect body weight and liver toxicity in mice inoculated with P3 cells a Body weight of mice treated with vehicle or Sim in combination with α-PD-1 and/or Lip-1. **b** and **c** Serum alanine aminotransferase (ALT) **b** and aspartate aminotransferase (AST) **c** levels in mice inoculated with B16 P3 cells and treated with the indicated reagents. For the *in vivo* experiments, 10 mice from each group were used. 5 samples were analyzed for **a**, and 3 samples were selected randomly for **b-c**. The p values by one-way ANOVA **a-c** are indicated**.** The error bars represent mean ± SD. ns, not significant. Source data are provided as a Source data file.

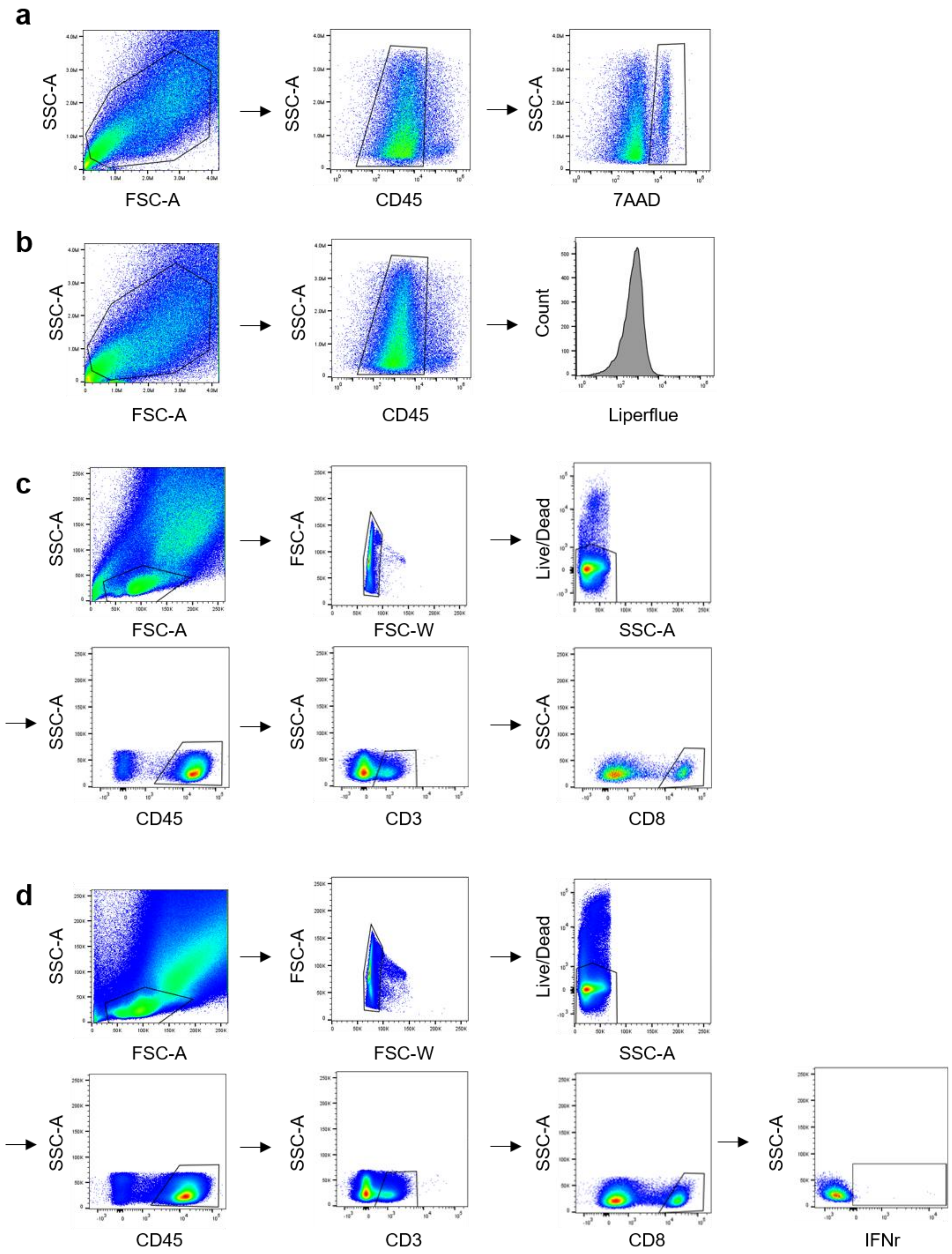

Supplementary Fig. 21 The gating strategy of flow cytometry analysis for in vivo tumor cell death a, lipid ROS b, T cell number c and cytokine production d.

Supplementary Fig. 22 uncropped images

Fig. 1g

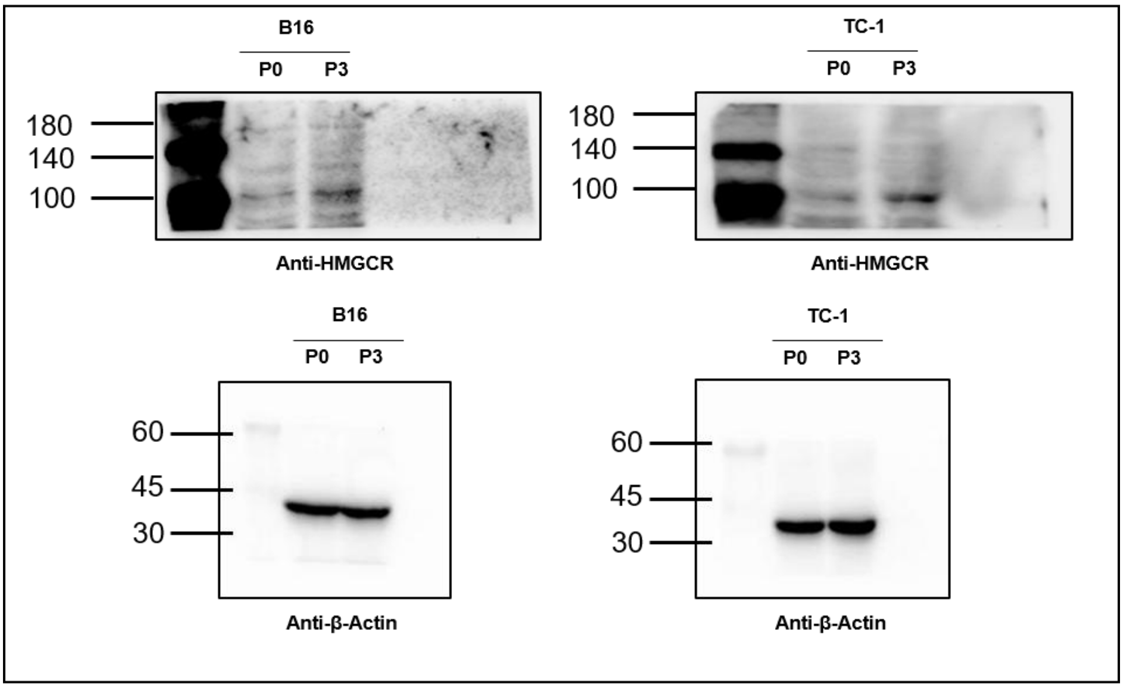

Fig. 3e

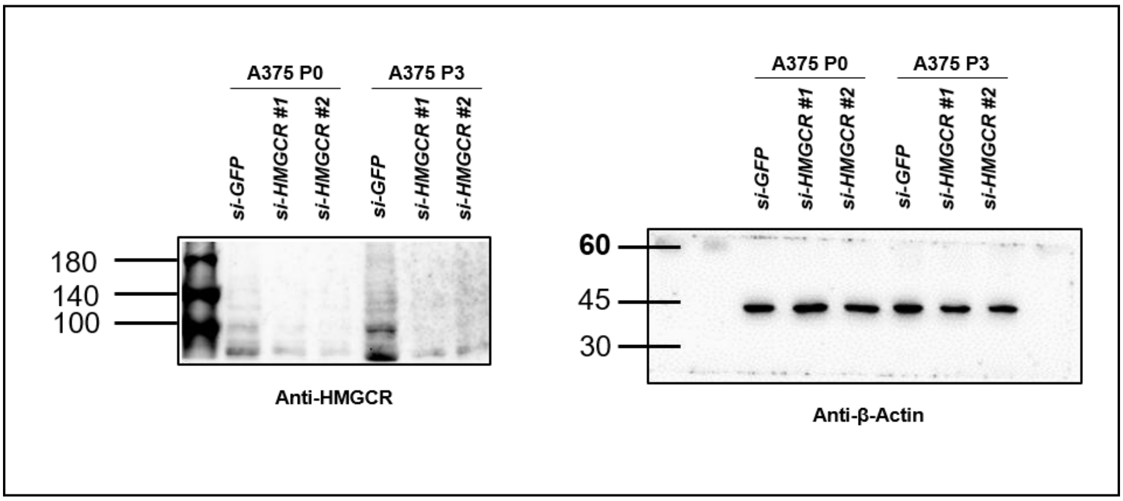

Fig. 4c

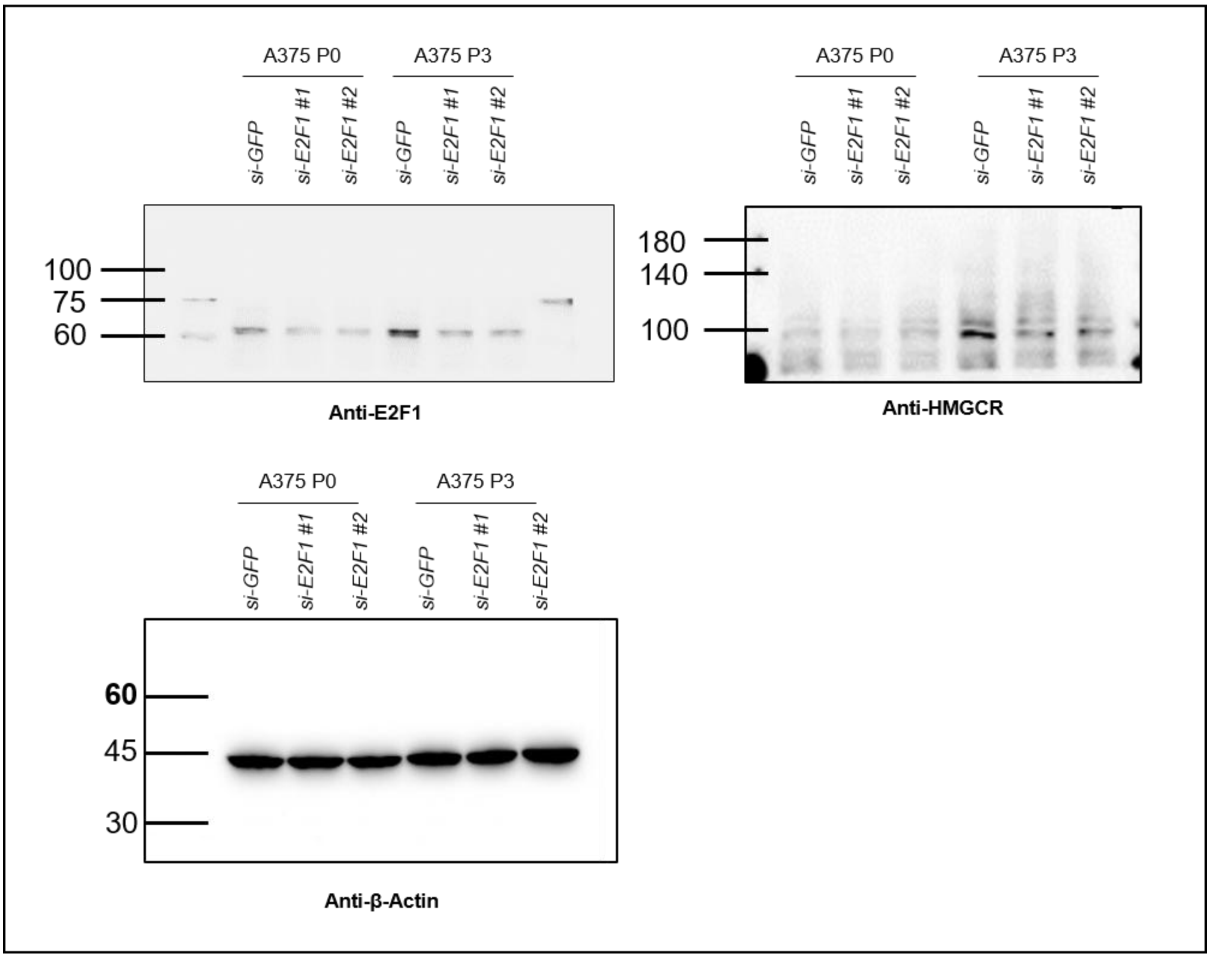
A375 P0
A375 P3
si-GFP
si-E2F1 #1
si-E2F1 #2
si-GFP
si-E2F1 #1
si-E2F1 #2
100
75
60
Anti-E2F1
A375 P0
A375 P3
si-GFP
si-E2F1 #1
si-E2F1 #2
si-GFP
si-E2F1 #1
si-E2F1 #2
180
140
100
Anti-HMGCR
A375 P0
A375 P3
si-GFP
si-E2F1 #1
si-E2F1 #2
si-GFP
si-E2F1 #1
si-E2F1 #2
60
45
30
Anti-β-Actin

Fig. 4k

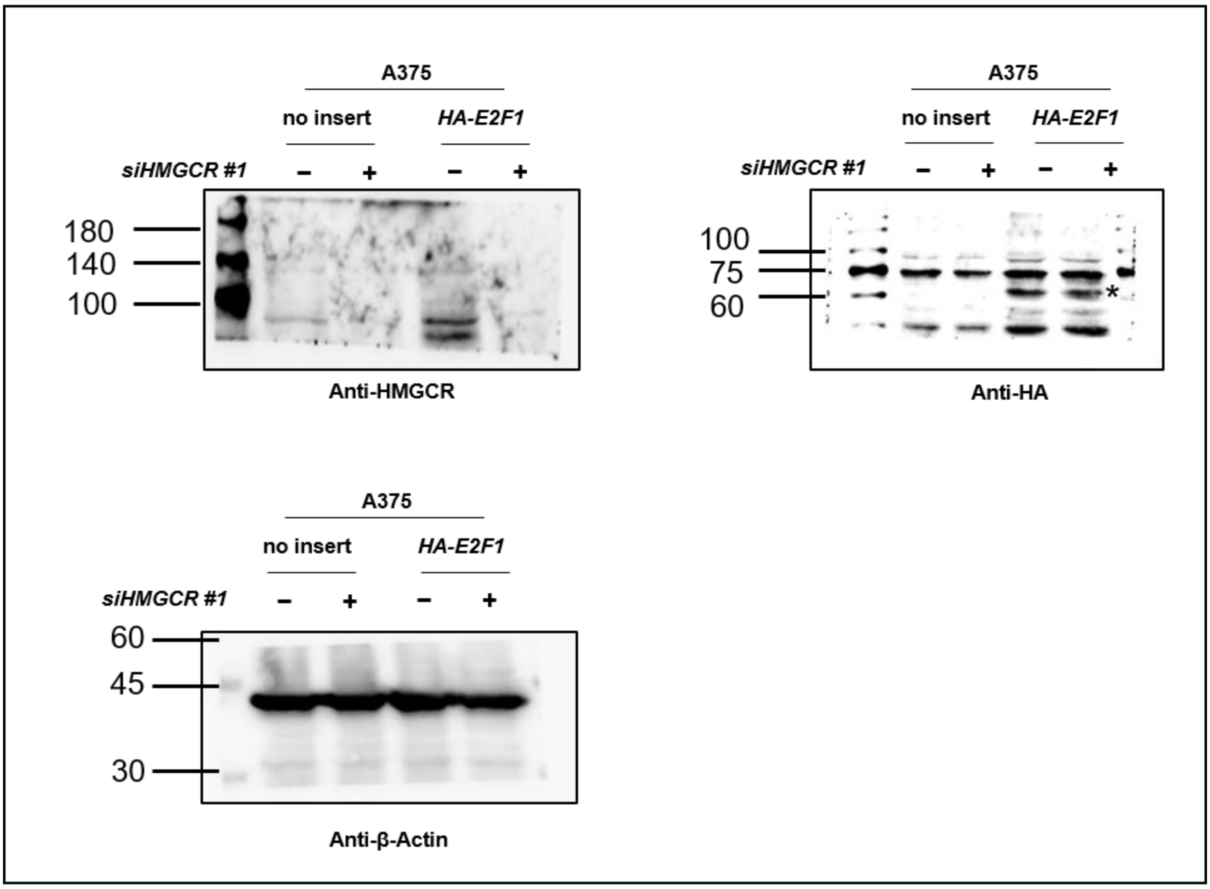

Supplementary Fig. 1e

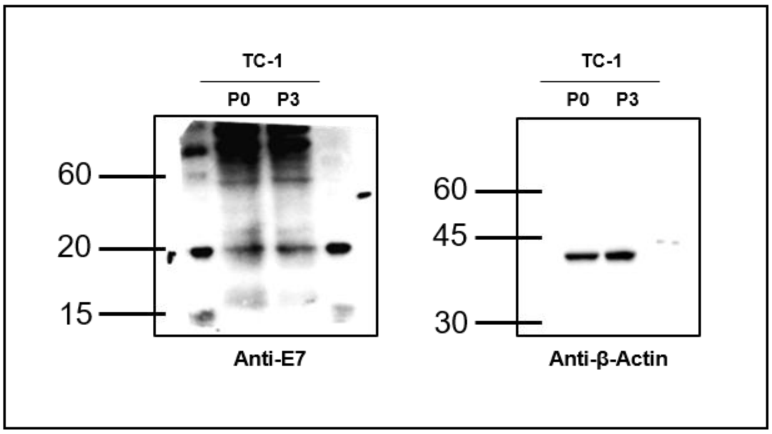

Supplementary Fig. 3a

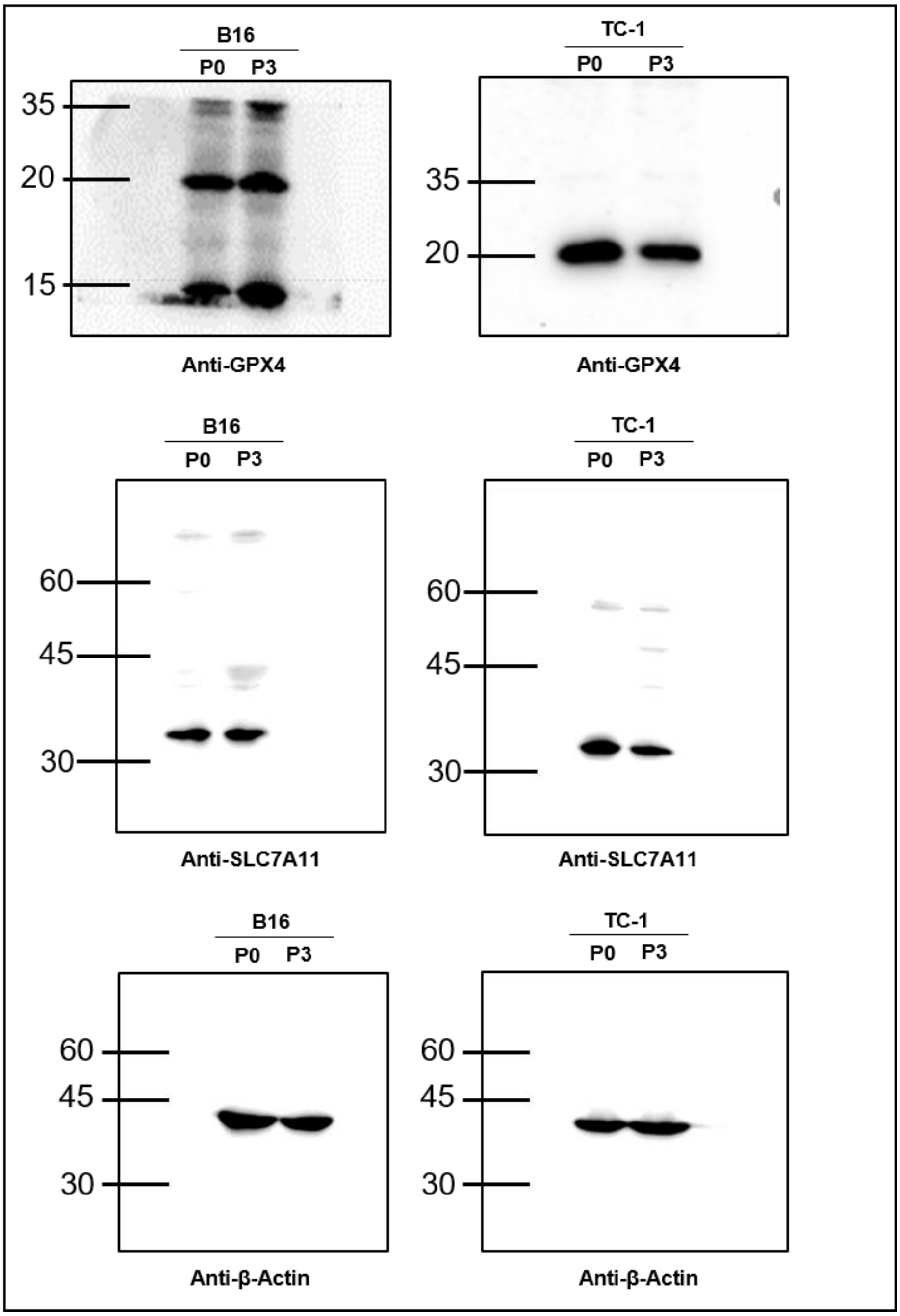

Supplementary Fig. 4a and 4b

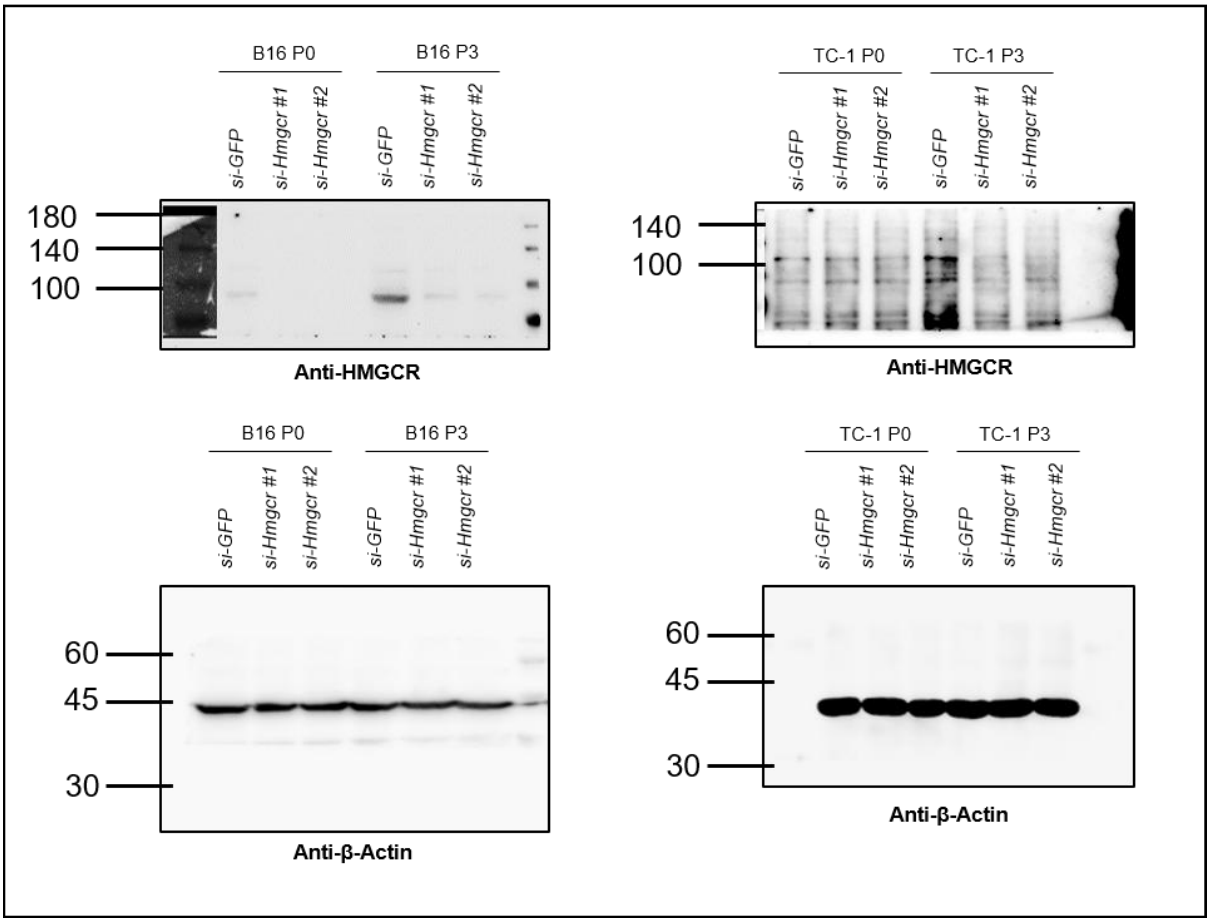

Supplementary Fig. 8a

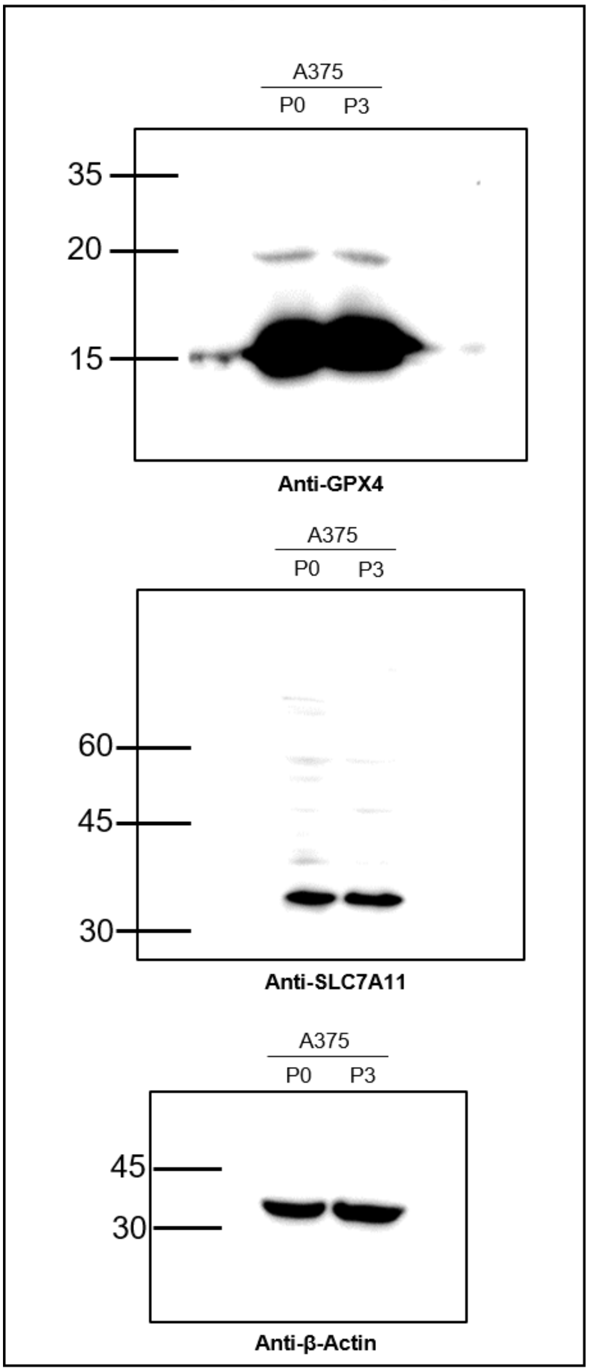

Supplementary Fig. 9a

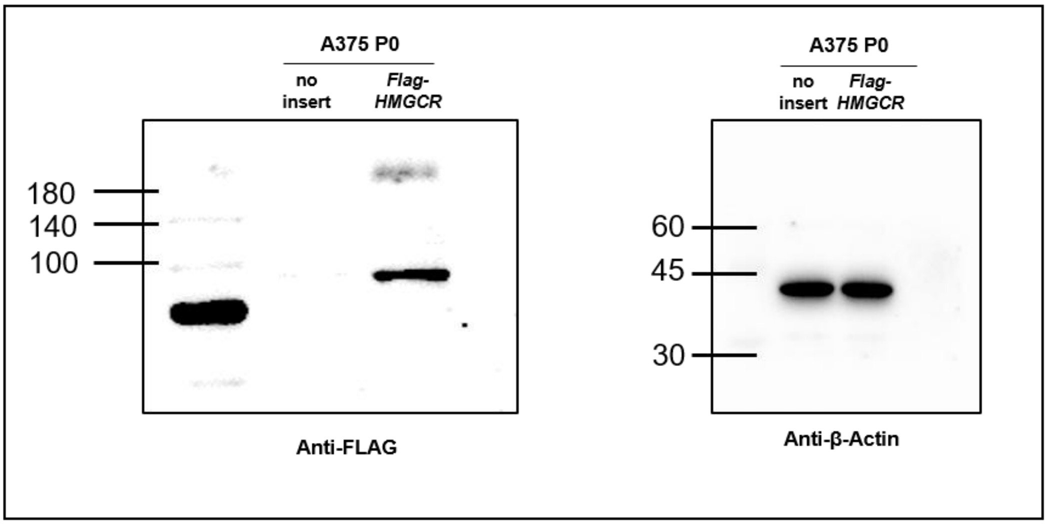

Supplementary Fig. 12a

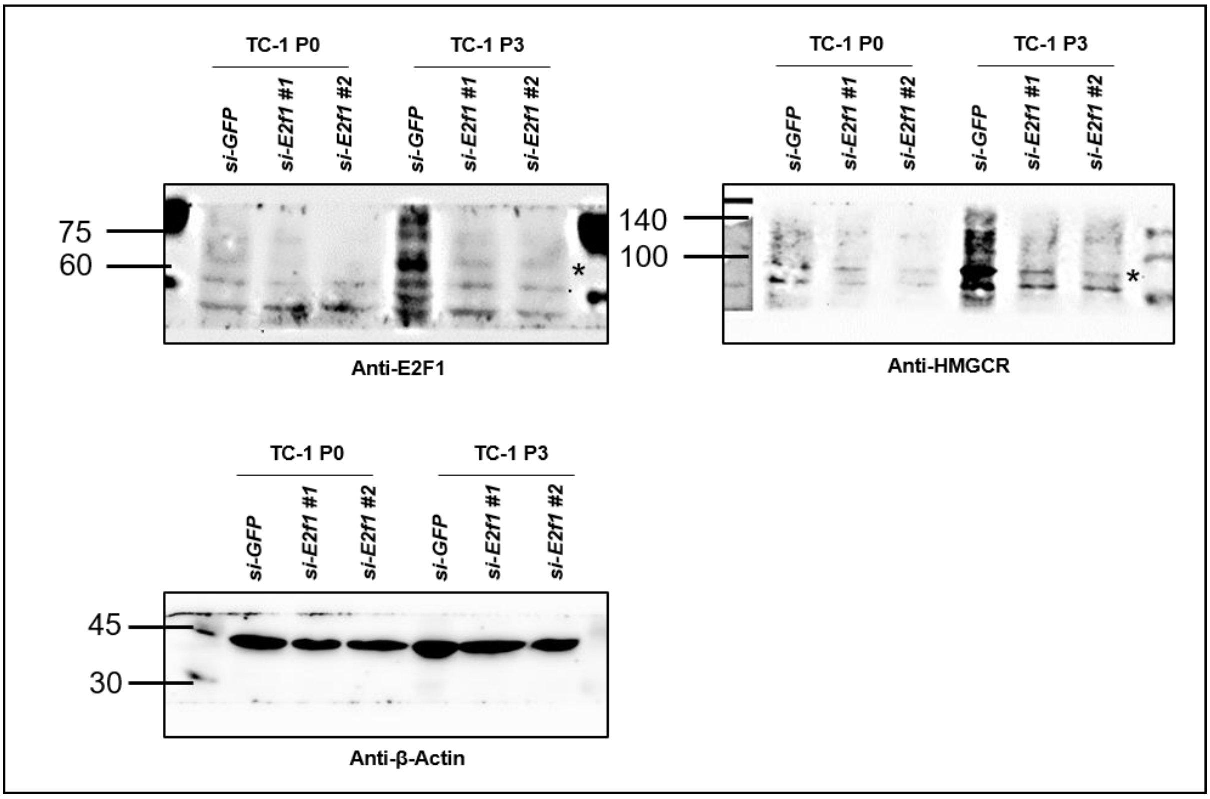

Supplementary Fig. 14b

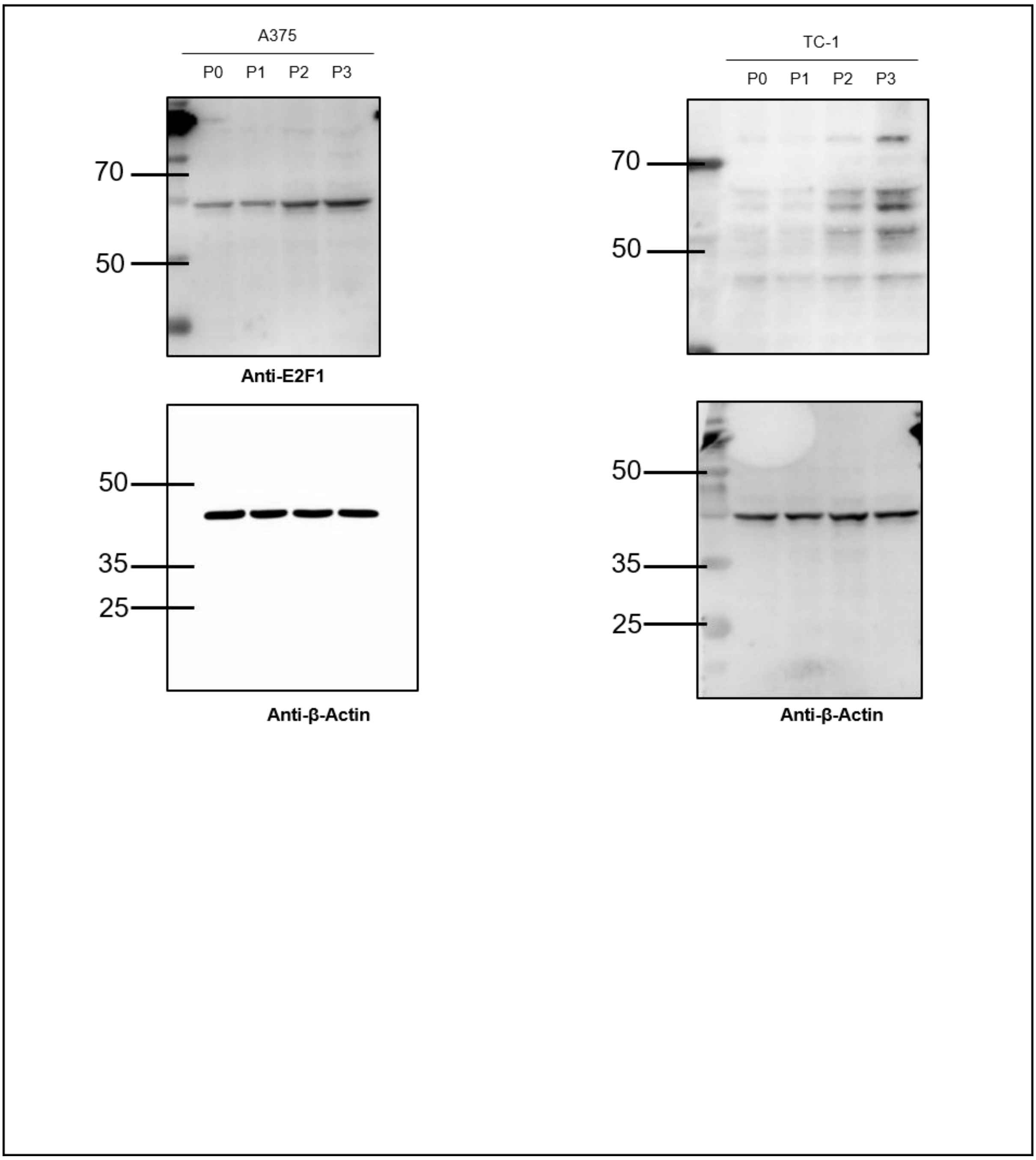

Supplementary Fig. 17b

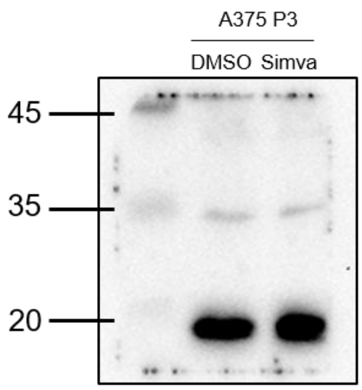

Anti-GPX4

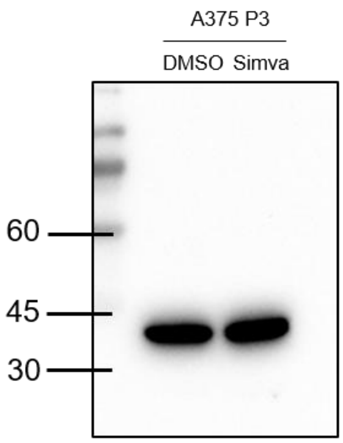

Anti-β-Actin

Supplementary Table S1: Primers used for quantitative RT-PCR

Primer name		sequence
hE2F1	F	5-ATGGTGATCAAAGCCCCTCC-3
	R	5-AAACATCGATCGGGCCTTGT-3
hHMGCR	F	5-CTTCCCAGCTTGTGTGTCCT-3
	R	5-GAGCTGCCAAATTGGACGAC-3
hSREBP1	F	5-TTGAAGCCTTCCTGAGCGG-3
	R	5-GCATGGACGGGTACATCTTCA-3
hSREBP2	F	5-GTACAGCCGGTCACCATTCA-3
	R	5-CCATTGGCCGTTTGTGTCAG-3
hFSP1	F	5-ATGGTGTTGCTACAGGGTGG-3
	R	5-AAACTTGCCAGGGAAGGGTC-3
hACTB	F	5-GAGCACAGAGCCTCGCCTTT-3
	R	5-ACATGCCGGAGCCGTTGTC-3
mHMGCR	F	5-AGCAGAGCTACGTTTGTGCT-3
	R	5-GGCTCGATGGGGAGTTCAAT-3
mSQLE	F	5-GTTCTGGTCAACCCCAGTCC-3
	R	5-TCTTGGCAATTCTCCCCGAA-3
mACACA	F	5-GGGAACATCCCCACGCTAAA-3
	R	5-GGCGTTGTCCAACAGAACATC-3
mSLC7A11	F	5-CCAAGGGCATACTCCAGAACA-3
	R	5-ACAGGGCTCCAAAAAGTGACA-3
mHMOX1	F	5-CCTCACAGATGGCGTCACTT-3
	R	5-GCTGATCTGGGGTTTCCCTC-3
mNQO1	F	5-GGCTGGTTTGAGAGAGTGCT-3
	R	5-CTGGAAAGGACCGTTGTCGT-3
mACLY	F	5-GAGGGGAAGCTGATCATGGG-3
	R	5-CTTGAGGATCTGCACTCGCA-3
mFDFT1	F	5-GCCGGCGGAATTTTATACCC-3
	R	5-AAAGCTGCGACTGGTCTGAT-3
mNRF2	F	5-AAAGCACAGCCAGCACATTC-3
	R	5-GGGATTCACGCATAGGAGCA-3
mFANCD2	F	5-TCCGAGAGGTTGTGAATGCC-3
	R	5-AGCCGACTAAGAACCTTGCC-3
mCISD1	F	5-GCCGATGCTGGAGGTCTAAA-3
	R	5-TCGCCAGTCTCTTCGTTGTG-3
mGPX4	F	5-CCGTCTGAGCCGCTTACTTA-3
	R	5-GGCTGAGAATTCGTGCATGG-3
mCBS	F	5-TTCAAGAGCTGAGCCTGTCG-3
	R	5-TGGTCAAAACCCTTCTCCCG-3
mHSBP1	F	5-TGAGCAGTCGGATTGACGAC-3
	R	5-GTCCAGTTCTTCTACTCCAGCC-3
mFTH1	F	5-GGAGCATGCCGAGAAACTGA-3
	R	5-TCTCCCAGTCATCACGGTCT-3
mACSL3	F	5-CGCCTTTTACTGTGTGGTGG-3
	R	5-GTCCATACCCCTGACCAACG-3
mACTB	F	5-TGCCCTGAGGCTCTTTTCCA-3
	R	5-GTTGGCATAGAGGTCTTTACGGA-3

Supplementary Reference

1 Derer, A. *et al.* Radio-Immunotherapy-Induced Immunogenic Cancer Cells as Basis for Induction of Systemic Anti-Tumor Immune Responses - Pre-Clinical Evidence and Ongoing Clinical Applications. *Frontiers in immunology* **6**, 505, doi:10.3389/fimmu.2015.00505 (2015).

2 Hong, X. *et al.* The Lipogenic Regulator SREBP2 Induces Transferrin in Circulating Melanoma Cells and Suppresses Ferroptosis. *Cancer discovery* **11**, 678-695, doi:10.1158/2159-8290.CD-19-1500 (2021).

3 Mishra, P. K. *et al.* Guidelines for evaluating myocardial cell death. *American journal of physiology. Heart and circulatory physiology* **317**, H891-H922, doi:10.1152/ajpheart.00259.2019 (2019).
