# Supplementary material for: The E2F1-HMGCR axis promotes ferroptosis resistance in immune refractory tumor cells
Source: Nat Commun. 2025 Dec 3;16:10849. doi: 10.1038/s41467-025-66282-x (PMC12675509; doi:10.1038/s41467-025-66282-x)
Supplement: Supplementary file 2 — Reporting Summary [file 41467_2025_66282_MOESM2_ESM.pdf]

nature portfolio

Corresponding author(s): Tae Woo Kim, and Kwon-Ho Song

Last updated by author(s): 18/9/25

Reporting Summary

Nature Portfolio wishes to improve the reproducibility of the work that we publish. This form provides structure for consistency and transparency in reporting. For further information on Nature Portfolio policies, see our Editorial Policies and the Editorial Policy Checklist.

Statistics

For all statistical analyses, confirm that the following items are present in the figure legend, table legend, main text, or Methods section.

n/a	Confirmed	
☐	☒	The exact sample size (n) for each experimental group/condition, given as a discrete number and unit of measurement
☐	☒	A statement on whether measurements were taken from distinct samples or whether the same sample was measured repeatedly
☐	☒	The statistical test(s) used AND whether they are one- or two-sided<br>*Only common tests should be described solely by name; describe more complex techniques in the Methods section.*
☐	☒	A description of all covariates tested
☐	☒	A description of any assumptions or corrections, such as tests of normality and adjustment for multiple comparisons
☐	☒	A full description of the statistical parameters including central tendency (e.g. means) or other basic estimates (e.g. regression coefficient) AND variation (e.g. standard deviation) or associated estimates of uncertainty (e.g. confidence intervals)
☐	☒	For null hypothesis testing, the test statistic (e.g. F, t, r) with confidence intervals, effect sizes, degrees of freedom and P value noted<br>*Give P values as exact values whenever suitable.*
☒	☐	For Bayesian analysis, information on the choice of priors and Markov chain Monte Carlo settings
☒	☐	For hierarchical and complex designs, identification of the appropriate level for tests and full reporting of outcomes
☒	☐	Estimates of effect sizes (e.g. Cohen's d, Pearson's r), indicating how they were calculated

Our web collection on statistics for biologists contains articles on many of the points above.

Software and code

Policy information about availability of computer code

Data collection	N/A
Data analysis	GraphPad Prism Ver. 10.0 (GraphPad Software, Inc., San Deigo, CA, USA), FACSuite software version 1.0.5. CytExpert software 2.4. FlowJo_v10.10.0. CHEA Transcription Factor Binding Site Profiles dataset https://maayanlab.cloud/Harmonizome/gene/HMGCR]

For manuscripts utilizing custom algorithms or software that are central to the research but not yet described in published literature, software must be made available to editors and reviewers. We strongly encourage code deposition in a community repository (e.g. GitHub). See the Nature Portfolio guidelines for submitting code & software for further information.

Data

Policy information about availability of data

All manuscripts must include a data availability statement. This statement should provide the following information, where applicable:

- Accession codes, unique identifiers, or web links for publicly available datasets
- A description of any restrictions on data availability
- For clinical datasets or third party data, please ensure that the statement adheres to our policy

RNA sequencing data have been deposited in the Gene Expression Omnibus (GEO) under the accession code GSE91061(Tumor and Microenvironment Evolution during Immunotherapy with Nivolumab, Cell (2017) Val 171, Issue 4, 934-949.e16). The source data underlying Figures 1-7 and Supplemental Figures 1-21 are provided in Source data file with this paper. All raw images for the immunoblots are provided in Supplemental Figure 22. The raw images for the immunoblots are provided in the supplementary information. All other data supporting the findings of this study are available from the corresponding author on reasonable request.

Research involving human participants, their data, or biological material

Policy information about studies with human participants or human data. See also policy information about sex, gender (identity/presentation), and sexual orientation and race, ethnicity and racism.

Reporting on sex and gender	N/A
Reporting on race, ethnicity, or other socially relevant groupings	N/A
Population characteristics	N/A
Recruitment	N/A
Ethics oversight	N/A

Note that full information on the approval of the study protocol must also be provided in the manuscript.

Field-specific reporting

Please select the one below that is the best fit for your research. If you are not sure, read the appropriate sections before making your selection.

[x] Life sciences [ ] Behavioural & social sciences [ ] Ecological, evolutionary & environmental sciences

For a reference copy of the document with all sections, see nature.com/documents/nr-reporting-summary-flat.pdf

Life sciences study design

All studies must disclose on these points even when the disclosure is negative.

Sample size	Sample sizes were pragmatically determined on the basis of the observed biological variability, and no formal statistical method was used to predetermine sample size. The exact n values for each experiment are provided in the figure legends
Data exclusions	No data were excluded.
Replication	All data are representative of at least 3 separate experiments. All attempt at replication were successful
Randomization	For in vivo treatment experiments, tumor-bearing mice were subjected to caliper measurements. Animals with comparable tumor sizes were randomized into treatment groups; this prevented outcomes from being influenced by initial differences in tumor burden. For in vitro cell experiments, all cells in each experiment were from the same parental cells.
Blinding	For the in vivo experiments, investigators were not blinded to group allocation because different treatments had to be administered to different groups. However, analyses of animal experiments were based on objectively acquired measurements, minimizing the risk of bias. For in vitro assays such as qRT-PCR, and Western blotting, blinding was not performed due to sample loading order, but the readouts were quantitative and objective. For flow cytometry and RNA-seq analyses, blinding was not relevant as the data acquisition was carried out by automated instruments. Overall, most of the experiments in this study were not applicable for blinding, and the absence of blinding did not affect the objectivity of the reported results

Reporting for specific materials, systems and methods

We require information from authors about some types of materials, experimental systems and methods used in many studies. Here, indicate whether each material, system or method listed is relevant to your study. If you are not sure if a list item applies to your research, read the appropriate section before selecting a response.

Materials & experimental systems

n/a	Involved in the study	
[ ]	[x]	Antibodies
[ ]	[x]	Eukaryotic cell lines
[x]	[ ]	Palaeontology and archaeology
[ ]	[x]	Animals and other organisms
[x]	[ ]	Clinical data
[x]	[ ]	Dual use research of concern
[x]	[ ]	Plants

Methods

n/a	Involved in the study	
[x]	[ ]	ChIP-seq
[ ]	[x]	Flow cytometry
[x]	[ ]	MRI-based neuroimaging

Antibodies

Antibodies used

Anti-active caspase-3 antibody (BD Pharmingen, Cat 561011, C92-605),
HMGCR (sc-271595, Santa Cruz Biotechnology, C-1),
HA (sc-805, Santa Cruz Biotechnology, Y-11),
FLAG (M185-7, MBL, FLA-1),
E2F1 (sc-251, Santa Cruz Biotechnology, KH95),
E7 (sc-6981, Santa Cruz Biotechnology, ED17),
GPX4 (52455T, Cell Signaling Technology, polyclonal),
SLC7A11 (12691T, Cell Signaling Technology, D2M7A),
CRT (Ab2907, Abcam, polyclonal),
β-ACTIN (sc-47778, Santa Cruz Biotechnology, C4),
anti-rabbit IgG-HRP (sc-2357, Santa Cruz Biotechnology, unkonwn clone),
anti-mouse IgG-HRP (Enzo, Cat ADI-SAB-100-J, polyclonal),
T cells including anti-CD45 (BD, #552848,k 30-F11),
anti-CD3 (BD, #555274, 17A2),
anti-CD8 (BioLegend, #100731, 53-6.7),
anti-IFN-γ (BD, #554412, XMG1.2),
cDC including CD11c (TONBO, #35-0114, N418),
CD103 (BioLegend, #121414, 2E7),
MHCII (BD, #553570, AF6-88.5) were used. For Western blotting, primary antibodies were used at a 1:1000 dilution and secondary antibodies at a 1:5000 dilution. For flow cytometry (FACS), antibodies were used at a 1:100 dilution. Lot numbers were not specifically recorded during the experiments, but all antibodies were commercially sourced and consistently used throughout the study.

Validation

Validation statements for antibodies can be found on their corresponding manufacturer datasheets and previous literature. All primary antibodies used in this study were validated according to the manufacturer's datasheets. Some of them have also been validated by our experiments, as shown in this manuscript, using either overexpression or knockdown strategies

Anti-active caspase-3 antibody (BD Pharmingen, Cat 561011, C92-605) https://www.bdbiosciences.com/en-ca/products/reagents/flow-cytometry-reagents/research-reagents/single-color-antibodies-ruo/pe-rabbit-anti-active-caspase-3.561011?tab=product_details,
HMGCR (sc-271595, Santa Cruz Biotechnology, C-1) https://www.scbt.com/ko/p/hmgcr-antibody-c-1,
HA (sc-805, Santa Cruz Biotechnology, Y-11) https://www.scbt.com/ko/p/ha-probe-antibody-y-11,
FLAG (M185-7, MBL, FLA-1) https://www.mblbio.com/bio/g/dtl/A/?pcd=M185-7,
E2F1 (sc-251, Santa Cruz Biotechnology, KH95) https://www.scbt.com/ko/p/e2f-1-antibody-kh95,
E7 (sc-6981, Santa Cruz Biotechnology, ED17) https://www.scbt.com/ko/p/hpv16-e7-antibody-ed17,
GPX4 (52455T, Cell Signaling Technology, polyclonal) https://www.cellsignal.com/products/primary-antibodies/gpx4-antibody/52455,
SLC7A11 (12691T, Cell Signaling Technology, D2M7A) https://www.cellsignal.com/products/primary-antibodies/xct-slc7a11-d2m7a-rabbit-mab/12691,
CRT (Ab2907, Abcam, polyclonal) https://www.abcam.com/en-us/products/primary-antibodies/calreticulin-antibody-er-marker-ab2907,
β-ACTIN (sc-47778, Santa Cruz Biotechnology, C4, https://www.scbt.com/ko/p/beta-actin-antibody-c4),
anti-rabbit IgG-HRP (sc-2357, Santa Cruz Biotechnology, unkonwn clone) https://www.scbt.com/ko/p/mouse-anti-rabbit-igg-hrp,
anti-mouse IgG-HRP (Enzo, Cat ADI-SAB-100-J, polyclonal) https://www.enzo.com/product/goat-anti-mouse-igg-polyclonal-antibody-ap-conjugate/,
T cells including anti-CD45 (BD, #552848,k 30-F11) https://www.bdbiosciences.com/en-us/search-results?searchKey=552848,
anti-CD3 (BD, #555274, 17A2) https://www.bdbiosciences.com/en-us/search-results?searchKey=555274,
anti-CD8 (BioLegend, #100731, 53-6.7) https://www.biolegend.com/en-us/products/percp-anti-mouse-cd8a-antibody-4256,
anti-IFN-γ (BD, #554412, XMG1.2) https://www.bdbiosciences.com/en-us/search-results?searchKey=554412,
cDC including CD11c (TONBO, #35-0114, N418) https://cytekbio.com/search?section=Products&q=35-0114,
CD103 (BioLegend, #121414, 2E7) https://www.biolegend.com/en-us/products/apc-anti-mouse-cd103-antibody-4914,
MHCII (BD, #553570, AF6-88.5) https://www.bdbiosciences.com/en-us/search-results?searchKey=553570

Eukaryotic cell lines

Policy information about cell lines and Sex and Gender in Research

Cell line source(s)

A375 (ATCC, CRL-1619, female, human melanoma) cell line was purchased from American Type Culture Collection (ATCC, Manassas, VA, USA). TC-1 cells were originally generated from C57BL/6 mouse lung epithelial cells transduced with HPV16 E6/E7 and Ras, female origin; kindly provided by TC Wu (USA, Johns Hopkins). Details about generation of TC-1 P3 cells lines used in this study are provided in the Materials and Methods section of the manuscript. B16 cells (mouse melanoma, female) were kindly provided by Jeong-Im Sin (South Korea, Kangwon National University). Generation of the immune edited B16 P3 cell line is described in The Journal of clinical investigation 2022;132. Generation of the immune edited A375 P3 cell line is described in Nature communications 2022;13, 2127.

Authentication

The identities of cell lines were confirmed by short tandem repeat (STR) profiling by IDEXX Laboratories Inc. and used within 6 months for testing.

Mycoplasma contamination	All cell lines were tested for mycoplasma using Mycoplasma Detection Kit (Thermo Fisher Scientific, San Jose, CA, USA) and negative for mycoplasma contamination.
Commonly misidentified lines (See ICLAC register)	No misidentified cell line was used in this study.

Animals and other research organisms

Policy information about studies involving animals; ARRIVE guidelines recommended for reporting animal research, and Sex and Gender in Research

Laboratory animals	6- to 8- week-old female NOD/SCID (NOD.CB17-Prkdcsscid /NCrKoat) or C57BL/6(C57BL/6NHsd) mice were purchased from KOATECH Inc. (http://www.koatech.co.kr) and used in this project. All mice were maintained in SPF1 (specific pathogen free-1) condition and cared by Korea University Institutional Animal care center policy. Mice were housed under a 12-hour light/dark cycle at an ambient temperature of 21–23 °C with relative humidity maintained at 40–60%.
Wild animals	This study did not involve wild animals.
Reporting on sex	The appropriate sex of mice was used in in vivo experiments according to the origin of each cancer cells. B16 P3, TC-1 and A375 female mice
Field-collected samples	The study did not involve samples collected from the field.
Ethics oversight	All mice were maintained and handled under the protocol approved by the Korea University Institutional Animal Care and Use Committee (KOREA-2017-0141). All animal procedures were performed in accordance with recommendations for the proper use and care of laboratory animals.

Note that full information on the approval of the study protocol must also be provided in the manuscript.

Plants

Seed stocks	N/A
Novel plant genotypes	N/A
Authentication	N/A

Flow Cytometry

Plots

Confirm that:

- [x] The axis labels state the marker and fluorochrome used (e.g. CD4-FITC).
- [x] The axis scales are clearly visible. Include numbers along axes only for bottom left plot of group (a 'group' is an analysis of identical markers).
- [x] All plots are contour plots with outliers or pseudocolor plots.
- [x] A numerical value for number of cells or percentage (with statistics) is provided.

Methodology

Sample preparation	For CTL-mediated apoptosis assay, tumor cells were labeled with CFSE (10 µM, Molecular Probes, Eugene, OR) in DMEM supplemented with 0.1% FBS. The CFSE-labeled tumor cells were pulsed with E7 peptide (10 µg/ml) for 1 hour if nessesary. The CFSE-labeled tumor cells were mixed with cognate tumor antigen–specific CD8+ CTLs at a 1:1 ratio and incubated for 4 hours at 37°C.<br>For CTL-mediated cell death assay, the A375 cells were incubated for 24 h with supernatant derived from NY-ESO1-specific CD8+ T cell lines. The frequency of ferroptotic cells was analyzed by staining with 7-aminoactinomycin D (7-AAD), active-caspase-3 or Liperfluo, and examined by flow cytometry<br>For cell death analysis, cells were treated with indicated reagents, collected, then resuspended in PBS containing 1 µg/ml 7-aminoactinomycin D (7-AAD), was obtained from Beckman Coulter. for 5 min, and directly run on a flow cytometer.<br>For Liperfluo staining, cells were treated with RSL3 in the absence or presence of liproxstatin-1 for an additional 20 h, Cells were stained in Hanks balanced salt solution (HBSS, Gibco) containing Liperfluo (10 µM) for 30 min at 37°C, collected by trypsinization, and analyzed immediately with a flow cytometer (Beckman coulter).<br>For immune cell tumor infiltration, treated mice were sacrificed on day 18 following tumor inoculation and tumors were

	harvested. Tumors were dissected into fragments by cutting, dissociated by a cell strainer. Cell suspensions were stained for intracellular and extracellular protein markers of interest.
Instrument	FACSVerse flowcytometer (BD Biosciences, Cat no. #651154, year 2014) and flowcytometer (Beckman coulter).
Software	Data analysis was performed in BD FACSuite and CytExpert software .
Cell population abundance	Purity was determined by running flow cytometry of the sorted population.
Gating strategy	Cell populations were first gated to exclude cell debris and aggregates based on FSC/SSC. Then cells stained with control were used to determine the boundary between "negative" and "positive" cells; this boundary was used to identify positive cells in samples stained with the specific antibody and reagent. The gating strategy is going to provide in Supplementary Figure 21

☒ Tick this box to confirm that a figure exemplifying the gating strategy is provided in the Supplementary Information.
